# Research on Rare Diseases in Germany – Using small fish and super-resolution microscopy to track down a rare disease

**DOI:** 10.25646/11834

**Published:** 2023-12-13

**Authors:** Nicole Endlich

**Affiliations:** Institute of Anatomy and Cell Biology, Greifswald University Medicine

**Keywords:** FOCAL SEGMENTAL GLOMERULOSCLEROSIS, PERSONALISED DIAGNOSTICS, ZEBRAFISH, SUPERRESOLUTION MICROSCOPY, PODOCYTES, MEDICATION

## Abstract

**Background:**

Focal segmental glomerulosclerosis (FSGS) is a rare disease, or damage to the filtering units of the kidney, the glomeruli, about of which there is only limited knowledge and few treatment options. The STOP-FSGS consortium has set itself the goal to expand our knowledge of this disease and develop new treatment options.

**Project:**

Through intensive research and the use of state-of-the-art techniques such as super-resolution microscopy, AI-based imaging and single-cell research, the consortium aims to gain a deeper understanding of the mechanisms of FSGS. This will allow the disease to be diagnosed more accurately and thus enable targeted and more effective treatment of patients. Another focus is on the search for drugs that slow down or even cure the disease.

**Results:**

By establishing a rapid animal model, i.e. zebrafish larva, potential substances/drugs were identified that can alleviate FSGS. Moreover, super-resolution microscopy was used to precisely quantify the structural changes in the kidney by determining the so-called ‘filtration slit density’ (FSD) and to identify a marker allowing a personalised prognosis and assessment of the course of the disease.

**Conclusions:**

The results obtained help to better recognise the progression of FSGS and to optimally adapt treatment in order to improve the quality of life of the afflicted individuals and avoid renal replacement therapies.

## Introduction

Focal segmental glomerulosclerosis (FSGS) is a rare and complex disease that involves damage to the kidney, in which certain areas of the glomeruli, i.e. of the blood-filtering units of the kidney, are damaged and scarred. In highly industrialised countries such as Europe, it manifests in around 20 individuals per million residents (adults) per year [1]. More than half of adult patients develop chronic or recurrent disease. In around 20 % of all children who require lifelong renal replacement therapy, the cause is FSGS [2, 3].

The exact causes and mechanisms of FSGS are not yet understood and may be multifactorial. Due to the lack of specific drugs or other therapeutic options [4], there is an urgent need for further research to deepen the understanding of FSGS and develop new treatment strategies. The acute phase is often treated with immunosuppressants (drugs that inhibit the immune system), particularly high-dose glucocorticoids, which have significant short- and long-term side effects. However, there has been little progress made regarding the therapeutic options and the patients’ mortality rate is 50 % within five years of the commencement of dialysis, regardless of age.

**Infobox Translational Research on Rare Diseases – a funding priority of the Federal Ministry of Education and Research**

A disease is considered rare if fewer than five in 10,000 people are affected by such a diagnosis. More than 8,000 rare diseases are known. It is estimated that more than four million people in Germany alone are affected by a rare disease.

Around 80 % of rare diseases are genetically determined, some diseases cause their first symptoms in childhood. However, the causes of the disease are often unexplored. The relatively small number of people affected, experts and suitable medicines complicate the path to a diagnosis and appropriate therapy. If there is no diagnosis or it can only be made at a late stage, irreversible courses of the disease are often a result. Therefore, research is vital for those affected. Basic research plays an important role here: it not only provides new insights into rare diseases, but can also contribute to a better understanding of more common diseases.

Since 2003, the Federal Ministry of Education and Research (BMBF) has been funding networks that jointly research causes and therapeutic approaches for rare diseases at various university locations. A coordination office supports these networks, among other things, in presenting their results to the public (see also https://www.research4rare.de/wp-content/uploads/2023/05/Poster_R4R_engl_2019-2026.pdf). At the European level, the research consortia are involved in the European Reference Networks (ERN) on rare diseases. In addition, there are international programmes, such as the European Joint Programme on Rare Diseases (EJP RD) for research into the diagnosis and therapy of rare diseases, in which the BMBF also participates.

FSGS leads to an impaired filtration function of the kidney, which is detectable by the formation of oedema and foamy urine. These are important indicators of reduced kidney function, which often leads to kidney failure. In severe cases, only lifelong dialysis or a kidney transplant can ensure the patient’s survival. Dialysis is extremely stressful for the patient and their relatives, though, and often leads to severe cardiovascular disease, which may be fatal.

In order to diagnose functional disorders at an early stage, i.e. before the kidney damage spreads broadly, novel and detailed analyses of the kidney tissue are needed.

However, as in the case of FSGS, this can be very challenging. A truly personalised analysis of tissue samples (biopsies) from the kidney using special markers, as well as measurable characteristics of the disease in the blood, are crucial for an early diagnosis and a prognosis of the course of the disease. The more precisely the disease pattern is analysed, the better the subsequent treatment can be. Advances in diagnostics may help to detect the disease at an early stage and optimise treatment.

In addition, it is important to identify drugs that can slow down or even stop the progression of the disease. This would significantly improve the quality of life of the afflicted individuals and reduce or even eliminate the need for invasive treatments such as dialysis or transplants and the associated risk factors.

FSGS Research is therefore of crucial importance in order to expand our knowledge of the disease and for the development of state-of-the-art procedures for personalised diagnostics and therapy.

## Project

The STOP-FSGS research network is an association of German FSGS experts. The methods used in the sub-project of the research network presented here were largely developed in the framework of the funding provided by the Federal Ministry of Education and Research (BMBF). These techniques can now be used for diagnostic procedures. Two methods in particular should be emphasised here: firstly, the method of quantitative analysis of kidney tissue based on super-resolution microscopy (PEMP: podocyte exact morphology measurement procedure) [5, 6] in combination with the use of new marker proteins (e.g. the claudin 5 protein) [7]. Secondly, high-throughput screening (automated mass testing of substances) using a zebrafish damage model was optimised and used for rapid identification of drugs [8, 9]. Aside from novel chemical substances, drugs that are already being used for other diseases also play a central role in a so-called ‘repurposing’ (utilisation of known active substances for a new purpose). This allows the drugs to be used more quickly in clinical application, as they already have a marketing authorisation.

## Results and classification

**Figure 1:**
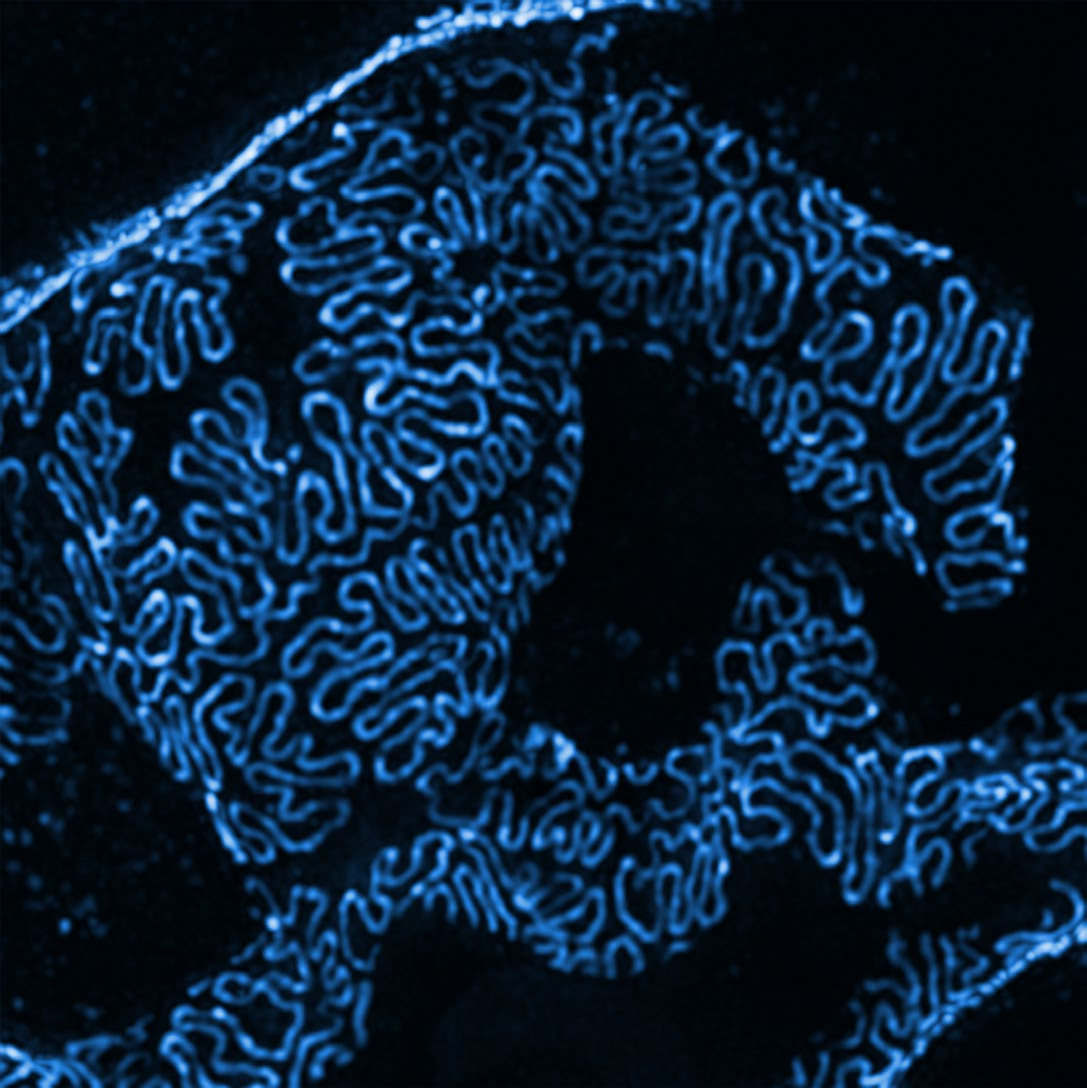
By using the super-resolution technique SIM, we can visualize the filtration slit, which is crucial for the proper functioning of the kidney. This method named PEMP can quantify changes in biopsies from patients as well as in animal models. The density of the bright lines is directly linked with the health of this kindey area, meaning the closer the lines are to each other, the healthier this part of the kidney is.

The STOP-FSGS project consists of several sub-projects, with our research area working on improving diagnostics through the use of super-resolution microscopy and on the identification of curative drugs. The super-resolution microscopy technology is a further development of light microscopy and received the Nobel Prize in 2014 [10]. In the STOP-FSGS project, this method was established for use in renal analyses. Using this method, biopsies of patients and experimental kidney samples from animals can now be quantitatively analysed for kidney diagnostics for the first time, and thus precise measurements of kidney changes can be made (Figure 1). This is a significant advance in kidney analysis, as the analysis of structural changes in the fine kidney structures could previously only be done with complex and non-quantitative methods, such as electron microscopy. The use of 3D super-resolution microscopy allows standard biopsies that are routinely carried out in pathology departments and research facilities now to be analysed rapidly, quantitatively and reproducibly. This is now used to calculate a new parameter named filtration slit density (FSD). For the first time, this provides exact values for the kidney changes and, in combination with a marker analysis, even renders a prognosis of the course of the disease possible. Current results demonstrate that this method enables personalised diagnostics for the first time, as precise measurements of the kidney changes associated with FSGS are can be obtained in both patients and laboratory animals [11–13].

In the second research area of the sub-project presented here, a group of substances with the potential to slow down or even stop the development of FSGS was discovered. Using high-throughput screening of zebrafish larvae, which are used as a model organism for FSGS, potential candidates were identified from 138 substances that have a protective effect in FSGS [14]. Due to the great similarity of the simple kidney of zebrafish larvae and human, rat and mouse kidneys, this model is well suited for the investigation of kidney diseases and identification of candidate drugs.

The results of this sub-project promise significant progress in FSGS research and might pave the way for more effective treatments in the future, some of which might also be transferred to other kidney diseases.

## Key statements

- Focal segmental glomerulosclerosis (FSGS) is a rare and complex disease or damage to the filter units of the kidney for which there are currently only limited treatment options.
- Using an established zebrafish model, candidate drugs for the treatment of FSGS have been identified.
- The high-resolution super-resolution microscopy allows for more precise diagnostics resulting in a personalised treatment of FSGS.
